# The Problems of Modern Psychiatry

**Published:** 1911-11-11

**Authors:** T. S. Clouston

**Affiliations:** late Physician-Superintendent Royal Asylum, Edinburgh, and Lecturer on Mental Diseases, University of Edinburgh, etc.


### THE PROBLEMS OF MODERN PSYCHIATRY.

An Introductory Lecture to the Class of Mental Diseases, University of Edinburgh.

By T. S. CLOUSTON, M.D., LL.D., late Physician-Superintendent Royal Asylum, Edinburgh, and Lecturer on Mental Diseases, University of Edinburgh, etc.

The study of mental diseases is one of extraordinary difficulty, but it is also one of the greatest interest. It has now a definite name in medicine: "Psychiatry." There are few departments of medicine where a narrow view of the subject would be more out of place than psychiatry. It is no doubt a specialty, but it is one where the very widest knowledge is necessary to its proper understanding. The mind of man is so incomparably the highest thing in the world, and the brain of man is so evidently the most complicated piece of organic machinery in nature, soundness of mind is so manifestly essential for human society, human work, and human law, that any defects of brain which affect the mind must be well worth our study. The conscious reference of every mental symptom to a brain change, the absolute correlation of the two in fact, is a new mental act to most physicians. Popularly looked at, the mind in the exercise of all its faculties, whether of consciousness, will power, judgment, feeling, or memory, is in no way necessarily connected with brain action at all. It is assumed that mind is self-existent, self-conditioned, self-controlled, and self-acting. The metaphysical and the religious ideas of mind have become automatic in civilised man, yet if one studies psychiatry with such a dominating idea it would be a mere mass of unintelligible facts.

We must go to anatomy, physiology, and modern psychology for the basis of mental action, and for the conditions under which it is exercised in the human being.

### The Cells.

We have as the primary and essential part of the brain—its cells; the number of these is enormous, probably about three thousand millions. Each cell of the larger type is in itself not a simple thing like a hepatic cell, but complicated to a degree almost past comprehension—its nucleus, its protoplasm, its chromatic material of varying amount, its interlacing fibres throughout its substance, encapsulating the nucleus and nucleolus, and continuing into the cell's processes. It undoubtedly possesses a most delicate cell-wall: its dendrites pass from it by the dozen and divide and subdivide into hundreds of branching fibres, each finished off by innumerable gemmules, mixing and in contact with the dendrites of other cells, if they are not organically connected with them. Then there is the all-important axion processes, which go to the organs of the body, so securing the passage of nerve energy to and from those organs. Those neurons, as the cell and its processes are now called, have suitable arrangements for absorbing from the blood the special material they require, this being chiefly done in sleep, and the instant removal by drainage of the used-up material is provided for in a very complete way.

In psychiatry our initial problem is to realise that every mental change and every mental disturbance is accompanied by, and may be said to be dependent on, the action of the neuron. There is an absolute psycho-physical parallelism between the cell action and the mental action. We can measure scientifically, or, at all events, we can imagine that such measurement can be made of the cell and every one of its energies except the mental. In all its relations except this we can imagine and we do believe there is an absolute relation of cause and effect—a "determinism" in everything connected with the cell action. The cell arises at first in embryo. It is slowly developed in structure and function. It slowly retrogrades and dies under fixed laws, but we must admit that in the case of the mental function of the cell, if we may call it so, we cannot subject it to measurement, or construct a scientific analysis of its laws, or bring it under any known physical laws of Nature. We can only assume that there is a definite parallelism—physiological and pathological. When we pass from physiological cell action to its mental concomitant or effect, we pass across an immeasurable gap and find ourselves in a new region. Herbert Spencer says that the scientific relation between organisation and thought is "unthinkable." Yet modern psychology, as distinguished from metaphysics, aided by experimental physiology, has tackled the problem of the exact relationships between mind and the general brain-work with some initial success.

The variety of form, and size, and number of dendrites of the cells of the brain is great. You know that there are at least six varieties of cell distinguished by the modern histologist, and even that number does not exhaust their variety. The so-called granular cells that lie on the very periphery of the brain convolutions, though so minute in size, are probably of extreme importance in the mental and other functions of the brain. It is certain that in the brain diseases like general paralysis, in which those granular cells undergo atrophy and change, mental disturbances invariably occur. It would be a mistake to think of the large, fully-organised cell, which forms the fifth layer in certain parts of the cortex, as being of really more essential importance than the granular cells or the other varieties; they are all equally necessary to right brain action. Dr. Campbell has recently given us studies of the brain cells in the various parts of the cortex, as the result of his exhaustive examination, that will be the groundwork of any right theory of their respective functions, but we know certain isolated facts only. We know that there are areas, more or less defined in the cortex, the functions of which differ. In idiocy, where mind is undeveloped, the brain cells are also undeveloped.

### The Fibres.

Though the neuron is undoubtedly the primary and main structure in the brain, to which every other structure is secondary, yet the nerve fibre is so essential a part of the organ in regard to its functions that it must be taken fully into consideration in realising the work of the brain as a whole. The function of the fibre is undoubtedly the transmission of nerve energy, just as the cable wire acts in the telegraphic system, transmitting the electricity generated in the batteries. The respective functional importance of the cell and the fibre is shown by the fact that the grey matter, which is chiefly cellular, receives five times as much blood as the white substance, which is chiefly fibrous. As yet a perfect map of the complications and connections of all the brain fibres cannot be made out. The two chief systems of fibres are, first, the *connective*, and, second, the *projection*. There are probably many subsidiary systems, such as the upward acting fibres from the basal ganglia up to the cortex. Also there are probably systems of fibres ever concentrating, enlarging, and diffusing the nerve action which originates in the brain. If Cajal's "Law of Avalanche" is a real mode of operation in the brain, slight stimuli, originating in one part of the brain, or coming from the outside, may increase in force and volume and so be delivered to their destination as very powerful stimuli indeed. An illustration of this is the case, frequently enough seen, of a very slight sensory impression setting up a severe neuralgia. It is very much allied to the motor symptom of a general convulsion following a slight local irritation in the brain. It is also probable that there is another kind of fibre whose function is exclusively inhibitory, and it is quite possible, I think, that there are fibres whose function is simply to dissipate energy into space, as it were, just like an earth-conducting wire in an electrical system. Overlaying the periphery of the whole cortex there is a minute network of very small unsheathed fibres which are evidently of the utmost importance as part of the connective system. When those undergo atrophy there are always mental defects.

The great thing to remember in psychiatry about fibres is that they connect every nerve cell with every other in the brain and every nerve group with every other nerve group; also that they connect the brain cortex, which is the seat of mind, with every tissue and every organ in the body. Hence the dominance of the brain cortex and its control over every organ and function. The general result of this most extensive series of connections is this—that the mental cortex has a suitable machinery through which it can act on every other system within the brain, that is, the motor, the sensory, the nutritive, and the vaso-motor systems, and also that it can act and be acted on by every organ and tissue in the body.

### Other Constituents of the Brain.

Though the other tissues in the brain are secondary to the nerve cell and fibre, yet their soundness and right working are essential to the right mental working of the cortex. The arterial system is a special one, the venous system is also quite special within the skull. The arterioles are different in their coats from those of any other part of the body, and the cortical capillaries have special characteristics of their own. The connective tissue, the neuroglia, is different from the ordinary connective tissue in the body, with a higher organisation and possibly even a relation to the nutrition of the cells and fibres. Disease of any of these accessory systems may interfere with the right mental working of the cerebral cortex.

In early non-development of the brain cortex just after or before birth we find the cells fewer in number, with a defective organisation, especially in respect to the number of dendrites. In certain brain diseases associated with mental disturbance we seem to have localised defects in certain varieties of the cells, as in epilepsy, but definite knowledge is still wanting on this all-important point.

### Ontogenetic and Phylogenetic Development and Heredity.

There is a close analogy between the development of the human brain from the third month after conception up to a year after birth (ontogenetic development) and that of animals low in the scale like the lizard up to the monkey (phylogenetic development). The same phases in the neuron are seen in both, the dendrites in the early child and in the lizard are the same simple structures with very few branches. There are histological appearances to be seen in the neurons in several forms of congenital imbecility, and even in mental disease, that closely resemble the normal dendrons of the lizard and frog. The "psycho-physical parallelism" of brain organisation and mental development is thus vividly seen.

### Flechsig's Discovery.

Flechsig's great discovery of the formation of new connective "nerve-paths" in the brain during its development is of the highest value as illustrating the importance of the fibre element in the brain. He found that from birth, as the various mental faculties are developed, special "nerve-paths" or strands of fibres are correspondingly developed, so that we have a mechanical means of explaining the association of various groupings of special cells with associated functions distant from each other. As the functions of sight and hearing are developed, fibres project themselves from the visual and the auditory centres to the mental parts of the brain, to the other sensory regions, and to the motor areas. Those are the first mental connections made, because mind is first roused into action through the senses. Thus sight leads to perception and to co-ordinated and associated muscular action. The whole brain as a machine is thus mechanically brought together into a harmoniously working organ.

While an exact scientific conception of the relation of mind to brain action is at present impossible, yet the new theory of the constitution of matter, namely, that instead of atoms we have to do with electrons, seems to give to the scientific imagination a basis which it had not before this theory was put forward. It seems certain that the arrangement and working of the electrons which constitute a mind cell must be vastly different from those which constitute a muscle rod or a liver cell.

*(To be continued.)*

